# Case Management Reduces Length of Stay, Charges, and Testing in Emergency Department Frequent Users

**DOI:** 10.5811/westjem.2017.9.34710

**Published:** 2018-02-12

**Authors:** Casey A. Grover, Jameel Sughair, Sydney Stoopes, Felipe Guillen, Leah Tellez, Tierra M. Wilson, Charles Gaccione, Reb J.H. Close

**Affiliations:** Community Hospital of the Monterey Peninsula, Division of Emergency Medicine, Monterey, California

## Abstract

**Introduction:**

Case management is an effective, short-term means to reduce emergency department (ED) visits in frequent users of the ED. This study sought to determine the effectiveness of case management on frequent ED users, in terms of reducing ED and hospital length of stay (LOS), accrued costs, and utilization of diagnostic tests.

**Methods:**

The study consisted of a retrospective chart review of ED and inpatient visits in our hospital’s ED case management program, comparing patient visits made in the one year prior to enrollment in the program, to the visits made in the one year after enrollment in the program. We examined the LOS, use of diagnostic testing, and monetary charges incurred by these patients one year prior and one year after enrollment into case management.

**Results:**

The study consisted of 158 patients in case management. Comparing the one year prior to enrollment to the one year after enrollment, ED visits decreased by 49%, inpatient admissions decreased by 39%, the use of computed tomography imaging decreased 41%, the use of ultrasound imaging decreased 52%, and the use of radiographs decreased 38%. LOS in the ED and for inpatient admissions decreased by 39%, reducing total LOS for these patients by 178 days. ED and hospital charges incurred by these patients decreased by 5.8 million dollars, a 41% reduction. All differences were statistically significant.

**Conclusion:**

Case management for frequent users of the ED is an effective method to reduce patient visits, the use of diagnostic testing, length of stay, and cost within our institution.

## INTRODUCTION

Frequent users of the emergency department (ED) represent a complex group of patients who overuse ED resources. This group accounts for as many as 28% of all ED visits, with the number of annual visits by this group continuing to rise.^1^–^4^ Frequent users of the ED are defined as patients making four or more ED visits per year; however, some “ultra”-frequent users may make 20 or more visits per year.^2^–^8^ It has been well established that ED frequent users increase healthcare costs and contribute to ED and hospital crowding.

While the reasons underlying frequent ED visits are often complex and may represent failure of the healthcare system to provide for patients with complex needs, ED frequent users incur significant charges and time for treatment and testing as a part of their evaluation and treatment. Additionally, as a part of each ED visit, evaluation, and treatment, patients spend time occupying EDs bed and using hospital services such as phlebotomy and radiology.^5^,^7^,^9^–^14^ ED bed time and hospital resources are a valuable commodity, particularly as ED visits continue to rise nationwide, making the reduction of such resources by ED frequent users a desirable goal.

Case management, as defined by the Case Management Society of America, is a “collaborative process of assessment, planning, facilitation, care coordination, evaluation, and advocacy for options and services to meet an individual’s and family’s comprehensive health needs through communication and available resources to promote quality, cost-effective outcomes.”^15^ Given the complex medical and social needs of ED frequent users, case management has been extensively used in this group of patients, with multiple studies showing successful reducing in the use of ED services and cost of care in the ED.^5^,^9^,^13^,^16^–^23^ A 2017 systematic review identified 31 different studies of interventions to decrease ED visits by frequent users.^19^ However, despite the large number of studies published, there has been little research on the effect of ED case management for frequent users on length of stay (LOS), either in the ED or in the inpatient setting. To the best of our knowledge, this is the first study to evaluate the effect of case management on ED, inpatient, and total hospital LOS for all types of visits by ED frequent users.

The goal of this investigation was to explore the effect of ED case management in frequent users of the ED on LOS, both in the ED and the inpatient setting. To better understand the impact of case management in this population, we also chose to look at the effect of this intervention on ED and hospital charges as well as utilization of hospital services. We hypothesized that ED case management would reduce ED visits, admissions, ED LOS, inpatient LOS, charges, and diagnostic studies.

## METHODS

We conducted this study at a 225-bed hospital in a suburban area, with approximately 56,000 ED visits per year. The surrounding healthcare community consists of a variable mix of county-run primary care clinics and private practice physicians – in both primary care and specialty care. There are few free clinics in the surrounding area. Two other hospitals are within 30 miles of our institution, one of which is a county hospital.

The study consisted of a retrospective chart review of ED and inpatients visits by patients in our hospital’s Emergency Department Recurrent Visitor Program (EDRVP), comparing the visits made in the one year prior to enrollment in the program, to the visits made in the one year after enrollment in the program. This study was considered exempt by our hospital’s institutional review board.

The EDRVP is run by an ED social worker or registered nurse (RN), with emergency physicians, social workers, ED RNs, chemical dependency providers, behavioral health RNs, case managers, and representatives from local insurance providers. At monthly meetings, members of the EDRVP discuss approximately 10 patients who have been referred to the program. If a care plan does not appear to be working to address frequent ED visits or a new issue has come up for the patient causing recurrence of heavy ED use, the patient’s case and care plan is re-visited at the next meeting. If a truly urgent or emergent issue arises, the staff will correspond via secure email or in person to address it and develop new care plans or revisions to existing care plans. The program was developed initially in 2006 by ED staff at our hospital to address increasing visits by frequent users. As the program has grown, additional hospital staff and services have been recruited to assist us with the growing number of patients requiring case management, and to meet newly identified needs of patients in the program.

**Population Health Research Capsule**

What do we already know about this issue?

Frequent users of the emergency department (ED) are high utilizers of healthcare resources. Case management has been proven to reduce the number of ED visits by frequent users of the ED.

What was the research question?

How does case management for ED frequent users affect ED and inpatient length of stay? Is the use of healthcare resources affected?

What was the major finding of the study?

Case management in this group reduced ED and inpatient length of stay. Admissions, testing, and hospital charges also decreased.

How does this improve population health?

Case management for ED frequent users can reduce over-utilization of healthcare resources by ED frequent users, allowing EDs to provide faster care to ED patients with normal ED use.

For inclusion criteria, patients are referred to the program for any of the following reasons: concerning ED use (as identified by an ED staff member); 10 or more ED visits in 12 months; six or more ED visits in six months; four or more ED visits in one month; or activity by a patient that demonstrates a propensity for future problematic ED encounters – such as violence in the ED or prescription forgery. Patients exhibiting such high-risk activity were believed to be potentially problematic patients, and therefore a plan was developed to preempt frequent, potentially dangerous, recurrent, and problematic visits. There are no exclusion criteria, and patients of any age may be referred. Once a patient has been referred for enrollment in the program, his or her visits are reviewed to determine the underlying medical, psychiatric, and social issues causing the multiple ED visits. A plan of care for the patient is then developed, with the intent to address these issues in the outpatient setting. Care plans may include referring the patient for a case manager, referring the patient to a needed specialist, assisting the patient with unstable housing, or requiring that patients only receive medications from their primary doctor – rather than coming to the ED for refills.

We studied all patients enrolled in the EDRVP between October 2013 and June 2015. For each patient, we reviewed all ED and inpatient visits for the one-year time period before they were enrolled as well as the one-year time period after they were enrolled. Visits were reviewed using the hospital’s electronic medical records system, Sunrise Clinical Manager (Version 14.3; Allscripts Healthcare Solutions. Chicago, IL). We recorded the number of each of the following parameters for the year before and year after enrollment: number of ED visits; number of inpatient admissions; ED LOS; inpatient LOS; ED charges; inpatient charges; number of computed tomography (CT) scans; number of ultrasounds; number of radiographs, and number of ED visits at which blood work was performed.

Additionally, we noted six main reasons why patients were referred to the program: needing pain management; complex psychosocial issues; complex medical conditions; psychiatric illness; substance abuse; and needing resources or referrals. We recorded the reason for referral for each of our patients. Six chart reviewers reviewed all of the visits and recorded the data using a standardized data collection spreadsheet in Microsoft Excel (Excel 2013; Microsoft Corporation. Redmond, WA). The lead author supervised the chart reviewers to ensure that data collection was standardized and accurate between them.

After data collection was complete, we proceeded with data analysis. As we wanted to determine the effect of ED case management on the study parameters listed above, we compared each of the parameters for each patient from the one-year time period before enrollment in the program to the one-year time period after enrollment in the program. To evaluate for statistical significance, we then used a paired Wilcoxon signed-rank test, comparing the year before enrollment to the year after enrollment. Statistical analysis was performed with Microsoft Excel and Max Stat (Version 3.60; MaxStat. Jever, Germany).

## RESULTS

Between October 2013 and June 2015, we enrolled 158 patients into the EDRVP program, which reflects our process of enrolling approximately 10 patients per month over this 19-month period. For administrative reasons, enrollment was significantly less than 10 patients per month on a few occasions. Demographic information of the patients can be found in Table 1. The oldest patient enrolled during this time period was 75 years old at the time of enrollment, with the youngest being nine months old at the time of enrollment.

In the one year prior to enrollment, patients in the program made 1,685 ED visits with 159 inpatient admissions, as compared to 855 ED visits with 97 inpatient admissions after enrollment. The number of CTs, ultrasounds, radiographs, and ED visits during which blood testing was done all decreased as well from the year prior to enrollment to the year after enrollment. All differences were statistically significant with a p-value of <0.05. The complete data on utilization of services is displayed in Table 2.

In the one year prior to enrollment, patients in the program occupied 125 days (a full 24-hour period) of ED bed time, along with 334 days of inpatient bed time, for a total of 459 days of ED and inpatient bed time. After enrollment in the program, this decreased to 83 days of ED bed time, 198 days of inpatient bed time, for a total of 281 days of ED and inpatient bed time. All differences were statistically significant with a p-value of <0.05. The complete data on LOS are displayed in Table 3.

In the one year prior to enrollment, charges incurred by ED visits by patients in the program were $5,827,162, with charges incurred during inpatient stays totaling $8,453,761, for a grand total of $14,280,923. In the one year after enrollment in the program, charges incurred by ED visits by patients in the program were $3,041,473, with charges incurred during inpatient stays totaling $5,405,175, for a grand total of $8,446,648. All differences were statistically significant with a p-value of <0.05. The complete data on charges are displayed in Table 4.

Finally, we reviewed the reasons that patients were referred to the program. The greatest number were referred for issues regarding substance abuse, and the need for improved pain management. Additionally, the majority of patients had more than one issue for which they were identified as needing assistance, with the average number of reasons for referral being two per patient. The complete data are displayed in Table 5.

## DISCUSSION

Our study clearly demonstrates that ED case management reduces utilization of services, LOS, and cost in a population of ED frequent users. Clearly in the current U.S. healthcare environment, which is characterized by expensive care and crowded hospitals and EDs, this is critical information and may provide some ideas to develop solutions to the problems of high cost and crowding. In reviewing the data on the reason for referrals to the program, it is apparent that this group of patients has complex needs, with less than a third of the group being referred to the program to address only one issue. This supports the need for a comprehensive case management program like the one we have instituted, as we believe that addressing only a single issue underlying recurrent ED use may not decrease ED utilization.

From an ED administration standpoint, the most compelling piece of data appears to be the effect of ED case management on LOS. EDs across the U.S. struggle with crowding, often with critically ill or injured patients being forced to wait in waiting rooms when no beds are available. Our study showed that ED case management for ED frequent users helps this problem in two ways. First, by reducing ED visits and ED LOS, the program directly decreases the amount of ED bed time occupied by these repeat visitors, freeing up beds for patients in the waiting room. Second, by reducing inpatient LOS, ED patients are more likely to have inpatient beds available when needed, reducing the frequency of ED boarding. With less ED boarding, there is more available bed time in the ED for new patients from the waiting room. This increased ability to place new patients from the waiting room allows for new patients to be roomed much more quickly, allowing for critically ill and injured patients to receive time-sensitive treatment more quickly and reducing the door-to-doctor time for all patients in the department.

In looking at the cost implications of our analysis, we must consider the payer mix when considering the implication of reducing ED and inpatient charges in such a drastic fashion, as insurance plans reimburse at variable rates. A 2016 Texas study found that for every $1.00 paid by Medicare to reimburse medical services, private insurance paid between $1.15 and $2.35, while Medicaid paid between $0.61 and $0.85.^23^ When looking at charges for services on the order of several million dollars, as in our study, the difference between reimbursement by private insurance and public insurance is enormous, also on the order of millions of dollars.

In our study, the majority of patients (57%) had Medicaid insurance, which (as demonstrated by the study above) results in lower reimbursements to the hospital as compared to other insurance programs. While we were unable to perform a formal cost analysis of the charges and reimbursements to the hospital due to limitations in access to the data, the fact that our intervention reduced visits predominantly by patients with Medicaid insurance is not likely to be financially harmful to the hospital. Furthermore, in reducing charges by the patients in our program, our intervention was able to save significant monies for all insurance programs in our healthcare system, which could be used for other health improvements and interventions, such as prevention and education.

Finally, it is clear that our intervention – case management for ED frequent users – decreased ED visits, with the results evident from our study, as well as multiple previous studies cited above. In our study, we noted a decrease in inpatient admissions, ED and inpatient LOS, charges, and the use of testing. The question arises as to whether case management reduces these metrics simply by keeping people out of the ED, or whether case management has some additional effect on utilization of services. In looking at Table 2, it becomes clear that ED visits decreased by 49%, with admissions and utilization of testing decreasing by about the same percentage, or slightly less. Continuing with Tables 3 and 4, LOS and charges decreased by less than 49%. This would suggest (although a formal analysis was not performed) that the most effective aspect of ED case management for frequent users is the ability to decrease ED visits, with all other decreased metrics the result of the patient not being in the ED (and therefore subjected to testing, charges, and possible admission).

## LIMITATIONS

Our study had several limitations. First, because we looked at ED and hospital visits at just one institution our study includes a relatively small number of patients. It is possible that patients in the program simply chose to seek care at other hospitals and EDs. Thus, while we were able to significantly reduce cost, LOS, and utilization at our hospital, similar parameters may have increased at neighboring hospitals due to patients avoiding our institution. A study of the effect of ED case management on multiple hospitals within a geographic region would provide valuable information on this issue.

Second, our study consisted of a retrospective chart review of a program in existence at our hospital, with no control group for comparison. While case management likely accounted for the significant changes in the parameters studied, it is possible that other factors, or simply regression towards the mean, accounted for part or all of our significant decreases.

Another limitation was that we did not look at testing utilization over the long term, but rather only compared the year prior to the intervention to the year after the intervention. For patients with recurrent complaints, physicians may not choose to perform imaging if imaging has recently been done. So, it is possible that robust imaging done on our patients in the year prior to enrollment decreased physician ordering of imaging studies in the year after enrollment. To be certain that our intervention decreased imaging study utilization, we would have needed to compare imaging in several years prior to enrollment to the year after enrollment.

Finally, as previously mentioned we did not conduct a formal cost analysis of charges and reimbursements to our institution to determine the impact of the significant reduction in ED charges. While again we speculated that with the majority of enrolled patients having Medicaid, the reduced charges represented savings to the hospital, it is possible that the program may have reduced reimbursements to the hospital in an unfavorable way.

## CONCLUSION

Case management is an effective means for reducing recurrent ED visits by frequent users. As a result of decreased ED visits, case management also was shown to reduce cost, length of stay, and utilization of testing – both in the ED and the inpatient setting.

## Figures and Tables

**Table 1:** Population in a study examining the effects of case management on frequent users of the emergency department. n = 158

	Total	Percent of total group
Homeless	12	7.6
Male	71	44.9
Female	87	55.1
Insurance		
Medicaid	90	57.0
Medicare	32	20.3
Tricare	3	1.9
Commercial	23	14.6
None	6	3.8
Other	4	2.5

Age at enrollment (mean) = 42.4 years

**Table 2:** Utilization of testing and services before and after enrollment of frequent ED users in a case management program.

	Pre-intervention	Post-intervention	Absolute change	Percent change	P-value
ED visits (1 year)	1685	855	−830	−49.26	<0.0001
Inpatient admissions (1 year)	159	97	−62	−38.99	0.002
Computed tomography	201	119	−82	−40.80	0.0001
Ultrasounds	71	34	−37	−52.11	0.01
Radiographs	384	239	−145	−37.76	<0.0001
ED visits during which blood testing was done	724	386	−338	−46.69	<0.0001

ED, emergency deparment.

**Table 3:** Length of stay (LOS).

	Pre-intervention	Post-intervention	Absolute change	Percent change	P-value
Length of stay (LOS) in minutes					
ED LOS	450041	299514	−150527	−33.45	<0.0001
Inpatient LOS	1204099	711671	−492428	−40.90	0.001
Total LOS	1654140	1011185	−642955	−38.87	<0.0001
Length of stay (LOS) in days					
ED LOS	125.01	83.20	−41.81	−33.45	<0.0001
Inpatient LOS	334.47	197.69	−136.79	−40.90	0.001
Total LOS	459.48	280.88	−178.60	−38.87	<0.0001

ED, emergency deparment.

**Table 4:** The change in charges (in U.S. dollars) before and after frequent users were enrolled in care management program.

	Pre-intervention	Post-intervention	Absolute change	Percent change	P-value
ED charges	5,827,162	3,041,473	−2,785,690	−47.81	<0.0001
Inpatient charges	8,453,761	5,405,175	−3,048,586	−36.06	0.003
Total charges	14,280,923	8,446,648	−5,834,275	−40.85	<0.0001

ED, emergency deparment.

**Table 5:** Reasons for referrals to Emergency Department Recurrent Visitor Program. n = 158

Reason for referral	Number of patients	% of total patients
Substance use	101	63.5
Need pain management	96	60.4
Psychiatric illness	46	28.9
Complex psychosocial issues	26	16.4
Needing resources/referrals	21	13.2
Complex medical conditions	20	12.6
Average number of reasons for referrals per patient		2
Number of reasons for referral		
Referred for 1 reason	47	29.7
Referred for 2 reasons	79	50.0
Referred for 3 reasons	23	14.6
Referred for 4 reasons	9	5.7
